# Amyloid-β Load Is Related to Worries, but Not to Severity of Cognitive Complaints in Individuals With Subjective Cognitive Decline: The SCIENCe Project

**DOI:** 10.3389/fnagi.2019.00007

**Published:** 2019-01-25

**Authors:** Sander C. J. Verfaillie, Tessa Timmers, Rosalinde E. R. Slot, Chris W. J. van der Weijden, Linda M. P. Wesselman, Niels D. Prins, Sietske A. M. Sikkes, Maqsood Yaqub, Annemiek Dols, Adriaan A. Lammertsma, Philip Scheltens, Rik Ossenkoppele, Bart N. M. van Berckel, Wiesje M. van der Flier

**Affiliations:** ^1^Department of Neurology and Alzheimer Center, Amsterdam University Medical Center, Vrije Universiteit Amsterdam, Amsterdam, Netherlands; ^2^Department of Radiology and Nuclear Medicine, Amsterdam Neuroscience, Amsterdam University Medical Center, Vrije Universiteit Amsterdam, Amsterdam, Netherlands; ^3^Amsterdam Neuroscience, Amsterdam, Netherlands; ^4^Department of Epidemiology and Biostatistics, Amsterdam Neuroscience, Amsterdam University Medical Center, Vrije Universiteit Amsterdam, Amsterdam, Netherlands; ^5^Department of Old Age Psychiatry, Amsterdam Neuroscience, GGZ inGeest, Amsterdam University Medical Center, Vrije Universiteit Amsterdam, Amsterdam, Netherlands; ^6^Clinical Memory Research Unit, Lund University, Malmö, Sweden

**Keywords:** subjective cognitive decline (SCD), Alzheimer’s diseaese, amyloid PET, self-awareness, early – biomarkers

## Abstract

**Objective:** Subjective cognitive decline (SCD) is associated with an increased risk of Alzheimer’s Disease (AD). Early disease processes, such as amyloid-β aggregation measured with quantitative PET, may help to explain the phenotype of SCD. The aim of this study was to investigate whether quantitative amyloid-β load is associated with both self- and informant-reported cognitive complaints and memory deficit awareness in individuals with SCD.

**Methods:** We included 106 SCD patients (mean ± SD age: 64 ± 8, 45%F) with 90 min dynamic [^18^F]florbetapir PET scans. We used the following questionnaires to assess SCD severity: cognitive change index (CCI, self and informant reports; 2 × 20 items), subjective cognitive functioning (SCF, four items), and five questions “Do you have complaints?” (yes/no) for memory, attention, organization and language), and “Does this worry you? (yes/no).” The Rivermead Behavioral Memory Test (RBMT)-Stories (immediate and delayed recall) was used to assess objective episodic memory. To investigate the level of self-awareness, we calculated a memory deficit awareness index (Z-transformed (inverted self-reported CCI minus episodic memory); higher index, heightened self-awareness) and a self-proxy index (Z-transformed self- minus informant-reported CCI). Mean cortical [^18^F]florbetapir binding potential (BP_ND_) was derived from the PET data. Logistic and linear regression analyses, adjusted for age, sex, education, and depressive symptoms, were used to investigate associations between BP_ND_ and measures of SCD.

**Results:** Higher mean cortical [^18^F]florbetapir BP_ND_ was associated with SCD-related worries (odds ratio = 1.76 [95%CI = 1.07 ± 2.90]), but not with other SCD questionnaires (informant and self-report CCI or SCF, total scores or individual items, all *p* > 0.05). In addition, higher mean cortical [^18^F]florbetapir BP_ND_ was associated with a higher memory deficit awareness index (Beta = 0.55), with an interaction between BP_ND_ and education (*p* = 0.002). There were no associations between [^18^F]florbetapir BP_ND_ and self-proxy index (Beta = 0.11).

**Conclusion:** Amyloid-β deposition was associated with SCD-related worries and heightened memory deficit awareness (i.e., hypernosognosia), but not with severity of cognitive complaints. Our findings indicate that worries about self-perceived decline may reflect an early symptom of amyloid-β related pathology rather than subjective cognitive functioning.

## Introduction

Amyloid-β plaques and neurofibrillary tangles are neuropathological hallmarks of Alzheimer’s disease (AD), which start to appear 10–20 years before the onset of dementia (Jack et al., 2013). Self-perceived cognitive decline in cognitively normal individuals is associated with a three- to six fold increased risk of AD (Schmand et al., 1996; Geerlings et al., 1999; Jessen et al., 2010). As such, a proportion of individuals with subjective cognitive decline (SCD) may harbor the earliest pathological changes associated with AD (Verfaillie et al., 2016, 2018a,b), particularly amyloid-β accumulation (i.e., preclinical AD) (Buckley et al., 2016; Perrotin et al., 2016; Hu et al., 2018).

Only a minority of individuals with SCD will develop AD within a few years (Slot et al., 2018a), but it is conceivable that individuals with preclinical AD exhibit a specific phenotype of cognitive complaints compared with individuals without underlying AD. There are many questionnaires to investigate the nature and severity of SCD, but the appropriate items enabling prediction of conversion to mild cognitive impairment (MCI) and dementia have not yet been identified (Rabin et al., 2015, 2017). There are various methodological challenges associated with SCD assessment, one of which is that cognitive complaints tend to vary as a function of demographic characteristics, such as level of education and age (Rabin et al., 2015). In addition, these factors can also act in synergy; for example, it has been shown that cognitive complaints in highly educated individuals are associated with increased risk of progression to AD, while this is not found in individuals with lower education levels (Jonker et al., 2000; van Oijen et al., 2007). SCD *plus* criteria were proposed in an effort to increase the likelihood of identifying preclinical AD in individuals with SCD. One of these criteria suggests that especially individuals who worry about their self-perceived cognitive decline are more likely to have preclinical AD, but associations between worries and amyloid-β load have not been confirmed in prospective studies yet (Geerlings et al., 1999; Jessen et al., 2010, 2014). Furthermore, former studies investigating associations between amyloid-β load and various SCD questionnaires have generated highly inconsistent results in cognitively normal individuals (Rodda et al., 2010; Amariglio et al., 2012; Mielke et al., 2012; Perrotin et al., 2012, 2016; Buckley et al., 2013; Holland et al., 2015; Snitz et al., 2015). These discrepancies could be due to the less precise amyloid-β positron emission tomography (PET) semi-quantitative cut-off values in preclinical stages of AD (Villeneuve et al., 2015), and variability of implemented SCD questionnaires, including the lack of informant reports or objective memory tests relative to self-reports (Rabin et al., 2015, 2017).

Another approach is to explore whether preclinical AD is linked to the insight in cognitive deficits (i.e., self-awareness) rather than to the severity of SCD. The degree of memory deficit awareness takes into account the contribution of objective memory performance or informant reports relative to self-reports of cognitive decline (Barba et al., 1995; Perrotin et al., 2015). A lack of awareness of memory deficits, anosognosia, is a striking symptom in patients with AD dementia (Barba et al., 1995; Perrotin et al., 2015). On the contrary, it is has been suggested that the earliest changes in cognition during preclinical stages of the disease are best perceived by the individual including a heightened sense of self-awareness for early brain changes (i.e., hypernosognosia) (Caselli et al., 2014; Langer and Levine, 2014). In a recent study self-awareness was defined as a discrepancy score between subjective and objective episodic memory performance and they found that cognitively normal individuals harboring amyloid-β pathology had a heightened sense of self-awareness (Vannini et al., 2017). It has, however, not yet been investigated whether the earliest changes in cognition are best perceived by the individual rather than the observer or objective memory tests in a memory clinic setting.

We hypothesized that increased amyloid-β load is related to specific cognitive complaints and heightened level of self-awareness. Therefore, the purpose of the present study was to investigate whether amyloid-β load, as measured using quantitative PET, may help to explain the phenotype of SCD in cognitively normal individuals who initially have been referred to a memory clinic. A second aim was to investigate whether amyloid-β load is associated with altered levels of self-awareness and informant reports of cognitive change.

## Materials and Methods

We included 106 SCD memory-clinic patients with [^18^F]florbetapir PET scans from the ongoing Subjective Cognitive ImpairmENt Cohort (SCIENCe) study (Slot et al., 2018b). Subjects were referred to our memory clinic by their general practitioner or medical specialist because of cognitive complaints. Prior to inclusion via the memory clinic, all patients underwent a standardized dementia screening according to the procedures of the Amsterdam Dementia Cohort (Van Der Flier et al., 2014). Screening included extensive neuropsychological assessment, physical and neurologic examination as well as laboratory serum tests (hemoglobin, thrombocytes, leucocytes, TSH, MCH, MCV, erycytes), and brain magnetic resonance imaging (MRI). A Dutch translation of the mini-mental state examination (MMSE) was used to screen for global cognition (Folstein et al., 1975). Clinical diagnosis was established by consensus in a multidisciplinary team. Individuals were labeled as having SCD when they presented with cognitive complaints, and results of clinical investigations were within normal range. Criteria for MCI, dementia, or any other neurological or psychiatric (e.g., major depression) disorders known to cause cognitive complaints were not met (Jessen et al., 2014; Molinuevo et al., 2017). In addition, we used the Hospital Anxiety and Depression Scale- Anxiety subscale (HADS-A) and Center for Epidemiological Studies Depression Scale (CES-D) scale to evaluate (subclinical) anxiety and depressive symptoms (cut-off ≥ 16), respectively (Radloff, 1977; Zigmond and Snaith, 1983). The study had been approved by the Medical Ethics Review Committee of the VU University Medical Center. All patients provided written informed consent.

### Image Acquisition and Analyses

Ninety minutes dynamic [^18^F]florbetapir PET scans were acquired on a PET/CT scanner (*n* = 59 on an Ingenuity TF and *n* = 47 on a Gemini TF, both from Philips Medical Systems, Best, Netherlands). PET images were corrected for attenuation, scatter, randoms, decay and dead time using standard software provided by Philips Healthcare. Three-dimensional T1-weighted MRI scans were co-registered to the PET scans, and regions of interest (Hammers template, *n* = 68 regions of interest [ROI]) were defined on the MRI scan (in native space) and superimposed onto the dynamic PET scan to obtain regional time activity curves using PVElab (Hammers et al., 2003; Rask et al., 2004). Receptor parametric mapping (RPM) with optimized settings (parameters settings 0.01–0.1, 50 basis functions) and cerebellar gray matter as reference region was used to generate images of binding potential (BP_ND_) relative to the non-displaceable compartment (Lammertsma and Hume, 1996; Gunn et al., 1997; Golla et al., 2018). From the BP_ND_ images, gray matter volume-weighted mean cortical BP_ND_ values were obtained. To investigate potential regional specificity, volume-weighted bilateral frontal, temporal (medial and lateral), and parietal cortical BP_ND_ values were also extracted. In addition, standardized uptake value (SUV, 50–70 min post-injection) images were visually assessed by a trained and experienced reader (BvB), leading to “normal” or “abnormal” classification of amyloid accumulation (for more details regarding visual reading of [^18^F]florbetapir PET images, please see https://www.accessdata.fda.gov/drugsatfda_docs/label/2012/202008s000lbl.pdf).

### SCD Assessment

We used four questionnaires with the following characteristics: two self-, one informant-based questionnaires, and one which was composed of five cognitive questions to assess SCD. The maximal time window between these assessments and the PET scan was 1 year (median = 3 months). We used both self- and informant reports of the Dutch translation of the Cognitive Change Index (CCI self and informant versions; each 20 questions [range 0–4], total score: 20–100) to assess cognitive function compared to 5 years ago (Rattanabannakit et al., 2016). We used the Subjective Cognitive Functioning (SCF, self-report) questionnaire (4 questions, range: –12 to +12) to assess self-experienced cognitive decline over a one-year time period.(Van Der Flier et al., 2014) SCF scores were inverted in such a way that higher scores reflect more complaints, comparable to the CCI. Finally, we used a structured patient interview to assess SCD. We used the following question “What complaints do you report?”. Based on the individuals’ spontaneous response the following cognitive domains were scored “yes/no”: memory, attention, organization, language, together with the follow-up question: “Does this worry you?” (Geerlings et al., 1999; Jessen et al., 2010). In addition, for descriptive purposes, the following question was used to inquire SCD onset “when was the first time that you talked with a physician about these problems?”

### Memory Self-Awareness Indexes

To investigate the level of self-awareness, two index scores were calculated. First, the memory deficit awareness index, was defined for each participant by calculating the difference between subjective and objective episodic memory scores (Barba et al., 1995; Perrotin et al., 2015; Vannini et al., 2017). In concordance with previous studies we used episodic memory (%remembered = [immediate/delayed recall] ^∗^ 100%) for the memory deficit awareness index, i.e., the Rivermead Behavioral Memory Test (RBMT)-Stories. To allow comparison between both measures, (1) the CCI-self was inverted in such a way that, similar to the objective memory score, a lower score indicated more severe subjective memory impairment; (2) both objective and the subjective memory scores were Z-transformed (Barba et al., 1995; Perrotin et al., 2015; Vannini et al., 2017). A positive index score reflects heightened self-awareness (hypernosognosia), whereas negative scores lowered self-awareness (anosognosia) (Barba et al., 1995; Perrotin et al., 2015; Vannini et al., 2017). To test the robustness of the memory deficit awareness index, we repeated the aforementioned procedures while using the Dutch version of the Rey auditory verbal learning test (RAVLT; immediate [5 trials summed] and delayed recall). Second, a self-proxy index (self-reported CCI minus informant-reported CCI) was calculated. A positive index score reflects more self-reported cognitive complaints than informant-reported complaints (hypernosognosia), whereas negative scores reflect more informant-based complaints than self-reported complaints (anosognosia).

### Statistical Analyses

Statistical analyses were performed using Statistical Package for the Social Sciences (SPSS, IBM v22). We used linear regression (for continuous outcome measures) or binary logistic regression (for dichotomous outcome measures) analyses to investigate associations between mean cortical [^18^F]florbetapir BP_ND_ (independent variable) and measures of SCD (i.e., CCI, SCF [total and single items scores], complaints questions). Analyses were adjusted for age, sex, education and depressive symptoms (CES-D). As cognitive complaints in highly educated individuals may be more predictive of dementia (Jonker et al., 2000; van Oijen et al., 2007), we also tested for an interaction education^∗^[^18^F]florbetapir BP_ND_. We repeated analyses for cortical lobar [^18^F]florbetapir BP_ND_. Linear regression analyses, adjusted for age, sex, education and depressive symptoms, were used to investigate associations between [^18^F]florbetapir BP_ND_ (independent variable) and memory self-awareness indexes (dependent variables; separate models). In addition, we also tested for an interaction education^∗^[^18^F]florbetapir BP_ND_. Associations were considered significant if *p* < 0.05.

## Results

**FIGURE 1:**
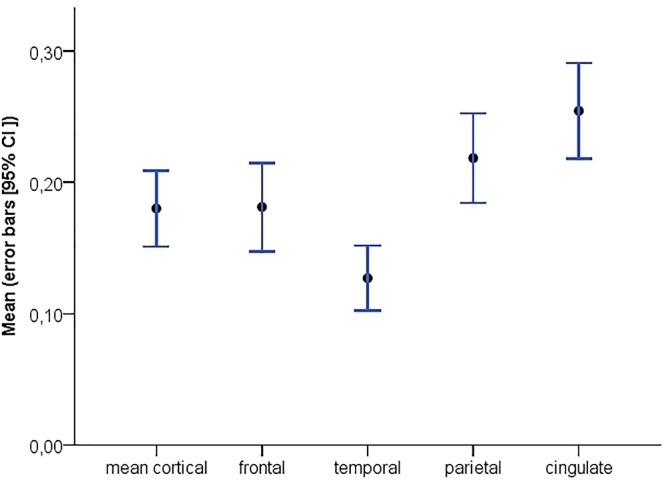
Distribution of global and regional [^18^F]florbetapir BP_ND_ with corresponding 95% confidence intervals.

Demographic and clinical data are presented in Table 1. Individuals (43% females) were (mean ± SD) 64 ± 8 years old and had an MMSE of 29 ± 1. Twenty-four individuals (23%) showed abnormal amyloid-β accumulation. On average, subjects had a mean cortical BP_ND_ of 0.18 ± 0.15 (Figure 1; frontal cortex 0.18 ± 0.18, temporal cortex 0.13 ± 0.13, cingulate cortex 0.25 ± 0.19, parietal cortex 0.22 ± 0.17). On average, individuals reported lower subjective cognitive functioning than 1 year earlier (SCF = –1.54 ± 2.92), and slight to occasional problems (CCI self-report: 41.23 ± 15.05; CCI informant report: 37.17 ± 16.44) compared to 5 years ago. There were no differences between self- and informant-based reports regarding the degree of cognitive change over a 5 year period (i.e., both CCI versions). About 68% (*n* = 73), 34% (*n* = 36), 13% (*n* = 14) and 25% (*n* = 27) of the individuals reported complaints in the domains of memory, language, organization and attention, respectively, whilst 47% (*n* = 50) felt worried about their self-perceived decline.

**Table 1:** Clinical and demographic data.

	Total group (*N =* 106)
**Demographics**
Male/female (*n*, %)	46/60 (43%M)
Age (years)	63.83 (7.65)
Education (range: 1–7)	5.79 (1.07)
SCD onset (% within last 5 years)	83%
Depressive symptoms (CES-D)	8.5 (7.0)
Anxiety (HADS-A anxiety subscale)	3.8 (3.2)
**Amyloid imaging**	
Net injected dose (MBq)	312 (38)
Specific activity (MBq/μg)	2.72 (1.76)
Visual assessment of SUV_50-70_ images (n abnormal [%])	24 (23%)
Mean cortical amyloid load ([^18^F]florbetapir BP_ND_)	0.18 (0.15)
Frontal cortex	0.18 (0.17)
Temporal cortex	0.13 (0.13)
Parietal cortex	0.22 (0.17)
Cingulate cortex	0.25 (0.19)
**Episodic memory**	
RBMT stories (version A+B) immediate recall	20.37 (5.94)
RBMT stories (version A+B) delayed recall	16.73 (6.20)
Rey auditory verbal learning test (RAVLT) immediate recall (5 trials)	45.36 (8.97)
Rey auditory verbal learning test delayed recall	9.28 (3.18)
**SCD questionnaires**	
SCF	–1.54 (2.92)
CCI self-reported	41.23 (15.05)
CCI informant-based	37.17 (16.44)
Memory question *n* “yes” (%)	73 (68%)
Attention question *n* “yes” (%)	27 (25%)
Organization question *n* “yes” (%)	14 (13%)
Language question *n* “yes” (%)	36 (34%)
Worry question *n* “yes” (%)	50 (47%)


*Data are presented as means (standard deviations) or percentages. Education level was assessed using the Verhage classification in accordance with the Dutch educational system. SCD onset was based upon individuals’ self-reports. Depressive symptoms were assessed with the CES-D*.

**FIGURE 2:**
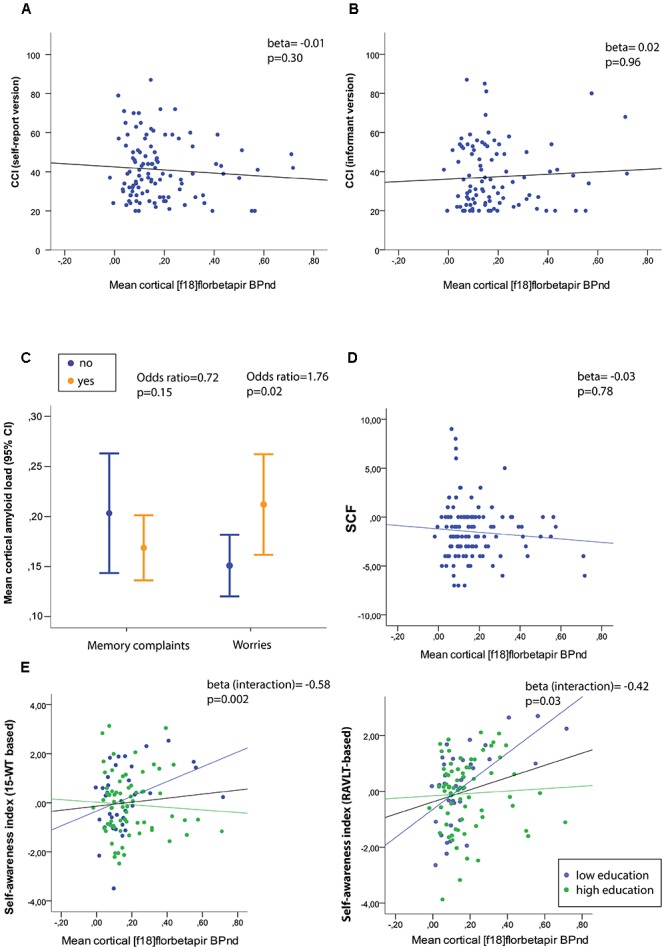
**(A)** Mean cortical amyloid-beta load in relation to raw (untransformed) scores of the self-report cognitive change index (CCI) and **(B)** informant-based CCI, and **(D)** the subjective cognitive functioning (SCF) questionnaire. **(C)** Mean cortical amyloid-beta load stratified for memory complaints (yes/no) and worries (yes/no). **(E)** Associations between mean cortical amyloid load and self-awareness index based on the RBMT % delayed recall (left) and RAVLT % delayed recall (right) with stratification for low and high education level. Memory deficit awareness index: A positive index score reflects heightened self-awareness (hypernosognosia), whereas negative scores lowered self-awareness (anosognosia).

Associations between mean cortical [^18^F]florbetapir BP_ND_ and measures of SCD are presented in Table 2. Adjusted for age, sex, education and depressive symptoms (CES-D), higher mean cortical (Figure 2) [^18^F]florbetapir BP_ND_ was associated with two-fold increased risk of SCD-related worries, but neither with any item nor total score of the SCF nor CCI (neither informant-based nor self-reported) nor dichotomous memory (Figure 2), attention, organization or language questions (all *p* > 0.05). There were no interaction effects between mean cortical [^18^F]florbetapir BP_ND_ and education for any of the SCD questionnaire outcomes (all *p* > 0.05). If we repeated analyses with cortical lobar [^18^F]florbetapir BP_ND_, we found that frontal (Odds ratio (OR) [95% confidence interval] = 1.70 [1.05–2.76]), cingulate (OR = 1.70 [1.06–2.73]), parietal (OR = 1.72 [1.08–2.74]), and temporal (OR = 1.86 [1.10–3.14]) cortical regions were associated with SCD-related worries, but not with other SCD questionnaires (all *p* > 0.05). Results remained essentially comparable when we repeated analyses while additionally adjusting for PET/CT scanner systems (data not shown).

**Table 2:** Associations between mean cortical amyloid-β load and SCD.

		*p*-values
**SCD questionnaires**
SCF	0.16 (–2.73 ± 1.92)	0.16
CCI self-reported	–0.10 (–10.01 ± 9.69)	0.30
CCI informant-based	0.01 (0.54 ± 10.38)	0.96
Memory question^∗^	0.72 (0.46 ± 1.12)	0.14
Worry question^∗^	1.76 (1.07 ± 2.90)	0.02
**Episodic memory**		
RBMT stories (% recall)	–0.16 (–1.09 ± 0.63)	0.09
RAVLT (% recall)	–0.09 (–0.62 ± 0.67)	0.35
**Memory self-awareness indexes**		
Self-awareness index (RMBT-based)	0.55 (4.82 ± 1.23)	<0.001
Interaction Education^∗^BPnd	–0.58 (–5.23 ± 1.61)	0.002
Self-awareness index (RAVLT-based)	0.38 (3.30 ± 1.27)	0.01
Interaction Education^∗^BPnd	–0.42 (–3.69 ± 1.65)	0.03
Self-proxy index	0.11 (0.71 ± 0.69)	0.31


*Data are presented as standardized Beta (unstandardized beta +standard error) or ^∗^odd ratios (95% confidence intervals). Analyses were adjusted for age, sex, education and depressive symptoms. Analyses between amyloid-β load, self-proxy and self-awareness indexes were based on mean cortical amyloid-β load. Self-awareness indexes; positive associations (i.e., betas) reflect heightened self-awareness (hypernosognosia) in relation with higher amyloid-β load, whereas negative scores reflect lowered self-awareness (anosognosia)*.

We furthermore investigated associations between mean cortical [^18^F]florbetapir BP_ND_ and two self-awareness indexes. We found that higher mean cortical [^18^F]florbetapir BP_ND_ was associated with a higher memory deficit awareness index (i.e., hypernosognosia) (Table 2 and Figure 2), with an interaction between BP_ND_ and education implying that this effect was stronger for individuals with relatively lower education (please see the full statistical models in Supplementary Table S1). When we repeated analyses with the memory deficit awareness index based on the RAVLT we found comparable results (Table 2 and Figure 2E right panel). There were no associations between mean cortical BP_ND_ and the self-proxy index, indicating that amyloid load was not related to discrepancy scores between self- and informant reports (based on the CCI).

## Discussion

The main finding of the present study is that amyloid-β load is associated with an approximately two-fold increased risk of SCD-related worries and a heightened memory-deficit self-awareness, but not with severity or specific cognitive complaints.

Amyloid-β load may insidiously affect cognition and self-perceived decline prior to symptom onset (Perrotin et al., 2012; Snitz et al., 2015; Baker et al., 2017), which could be amongst others a reason for individuals to visit a memory clinic. Some population-based and mixed population and memory clinic studies have shown that amyloid-β load is related to SCD (Amariglio et al., 2012; Mielke et al., 2012; Perrotin et al., 2012, 2016; Snitz et al., 2015), but other studies did not find this association with SCD (Buckley et al., 2013; Holland et al., 2015). To the best of our knowledge, associations between amyloid-β load and cognitive complaints have not been investigated in a pure memory clinic sample, and earlier findings could have been affected by the recruitment policy, particularly in the case of mixed recruitment studies (Jansen et al., 2015; Slot et al., 2018a). Therefore, it remains unclear to what extent amyloid-β is contributing to the phenotype of SCD in individuals who seek medical evaluation for their complaints (Jonker et al., 2000; Stewart, 2012). In the present study we investigated relatively young individuals with a recent SCD onset (<5 years), and compared to literature (Jansen et al., 2015; Ossenkoppele et al., 2015), a substantial fraction of almost one out of four showed abnormal amyloid-β accumulation. We furthermore used quantitative PET (i.e., BP_ND_) because it can more accurately determine amyloid-β load than standardized uptake ratio values (SUVr), which is especially important to capture potentially early – subtle – disease processes, and SCD-related worries are related to a sixteen and six fold increased risk of clinical progression, respectively (Jessen et al., 2010; Van Harten et al., 2013; Buckley et al., 2016), and we now show that they associated with each other in cognitively normal individuals. The SCD *plus* criteria have suggested that the presence of SCD-related worries are associated with an increased risk of future cognitive decline (Jessen et al., 2014), and our results support this notion.

Apart from the relationship with SCD-related worries, no associations between amyloid-β load and any of the SCD questionnaires or single items that measure various aspects of cognitive change were observed, indicating that these questionnaires are not specific for amyloid-β accumulation in a memory clinic. There is much controversy about which questionnaire can be used to unveil cognitive normal individuals at increased risk for AD, but the present results indicate that it does not necessarily matters which questionnaire, but rather whether the SCD assessment includes a “worry” inquiry. Earlier studies have used SUVr to assess abnormal amyloid-β accumulation, which have generated inconsistent results (Mielke et al., 2012; Perrotin et al., 2012, 2016; Holland et al., 2015; Snitz et al., 2015). Compared with BP_ND_, SUVr is liable to overestimation of amyloid-β load together with a higher variability (van Berckel et al., 2013), which could hamper a correct interpretation and reduce statistical power especially in early disease stages (Yaqub et al., 2008; van Berckel et al., 2013; Golla et al., 2018). Other possible explanations for our findings are recruitment criteria used and the operationalization of SCD (Perrotin et al., 2016; Molinuevo et al., 2017). A recent study has demonstrated elevated levels of amyloid-β load in memory clinic SCD patients compared with community-dwelling individuals without SCD, but not compared with community-recruited subjects with SCD (Perrotin et al., 2016). The present investigations were restricted to individuals with SCD, who had visited a memory clinic for their self-perceived decline. By definition these individuals experience cognitive complaints, but these may be caused by various factors other than amyloid-β accumulation. For example, it has been shown that memory clinic SCD patients have higher (subclinical) depressive symptoms compared with community-dwelling individuals with SCD (Perrotin et al., 2016). In the present study, however, individuals with a current psychiatric diagnosis were excluded prior to enrolment. In addition, analyses were adjusted for depressive symptoms, which makes it unlikely that mental illness was responsible for the lack of associations. Nevertheless, irrespective of the nature of cognitive complaints, higher mean cortical amyloid-β load was associated with SCD related worries, and this appeared to be consistent for all cortical lobar regions.

It has been claimed that earliest changes in cognition are best perceived by the individual rather than by an observer (Caselli et al., 2014). In order to investigate the awareness of memory deficits, two discrepancy scores were calculated to adjust cognitive complaints for episodic memory performance (i.e., memory deficit awareness index) and informant reports (i.e., self-proxy index). Although SCD conceptually refers to the self-perception of cognitive decline and does not require confirmation by informants, we did not find different associations between amyloid-β and the self-proxy index. In line with a study on cognitively normal individuals from the community (Vannini et al., 2017), we found a positive relation between amyloid-β load and memory deficit awareness, which indicated that individuals with increased amyloid-β load showed heightened memory deficit awareness or hypernosognosia. We furthermore found that associations were dependent on the level of education. The previous indicates that when taking into account the degree of delayed memory recall with cognitive complaints this could result in a stronger relationship with amyloid-β load, particularly for individuals with relatively lower education levels. Earlier studies have used this index to investigate anosognosia, and showed that AD patients have an impaired memory deficit awareness, i.e., more severe episodic memory performance compared with self-rated cognitive performance (Barba et al., 1995; Perrotin et al., 2015). In the present study we found opposite patterns compared to patients with AD dementia (Perrotin et al., 2015). Although it needs to be interpreted with care, these positive associations could reflect a higher level of memory deficit self-awareness in individuals with elevated amyloid-beta accumulation (Vannini et al., 2017). The index scores seemed to be slightly driven by episodic memory performance, and our findings imply that higher amyloid-β load can be observed in cases when individuals’ self-rated cognitive complaints are less severe than their episodic memory performance, which is in line with studies showing that episodic memory starts to deteriorate early in the disease course (Hanseeuw et al., 2017; Lim et al., 2018).

Although individuals with SCD who visit a memory clinic are a clinically relevant group since they seek help for their complaints and are at increased risk for clinical progression (Geerlings et al., 1999; Jonker et al., 2000; Jessen et al., 2010), some limitations need to be acknowledged. First, while we have incorporated every available SCD item from our cohort, there may be other questionnaires which are better able to isolate SCD due to preclinical AD. On the other hand, the use of other SCD questionnaires may not provide very different results, because questionnaires will likely show high correlations, and typically inquire about memory complaints (Rabin et al., 2015). In addition, questionnaires rely on self-perception of cognitive decline, which in the present study did not show any relation with amyloid-β load or could have been distorted by other non-AD or subthreshold psychiatry SCD phenotypes (Buckley et al., 2015; Slot et al., 2018b). Notwithstanding, our results indicate that not SCD severity, but rather worries about self-perceived decline can be relevant. Second, we have not included individuals without cognitive complaints, therefore we cannot extrapolate our findings to the general population. On the other hand, we do expect that individuals who feel worried about their cognitive decline will most likely visit a memory clinic. Third, our memory self-awareness index seemed driven by relatively lower, but non-significant, episodic memory performance in individuals with higher amyloid-β burden. Lastly, the present study had a cross-sectional design. Therefore, it is not possible to make inferences as to whether individuals with more severe amyloid-β accumulation and SCD related worries will show an increased risk of clinical progression to symptomatic stages of AD. Future longitudinal studies are necessary to fully elucidate these associations, while taking into account the effects of concomitant AD pathology such as tau burden (measured with PET, CSF or blood) and hippocampal atrophy.

In conclusion, amyloid-β load was associated with SCD related worries and higher memory deficit self-awareness (i.e., hypernosognosia), but not with severity or specific pattern of cognitive complaints. Our findings indicate that worries about self-perceived decline may therefore help to identify amyloid-β related SCD.

## Author Contributions

SV acquired, analyzed, and interpreted the data, drafted the manuscript, and approved the final content of the manuscript. TT, CvdW, LW, and RS acquired the data, critically revised the manuscript, and approved the final content of the manuscript. RO, SS, NP, AD, MY, BvB, and AL conceived and designed the study, analyzed and interpreted the data, drafted the manuscript and enhanced its intellectual content, and approved the final content of the manuscript. PS and WvdF conceived and designed the study, enhanced the intellectual content of the manuscript, and approved the final content of the manuscript.

## Conflict of Interest Statement

The authors declare that the research was conducted in the absence of any commercial or financial relationships that could be construed as a potential conflict of interest.
