# Supplementary material for: Amyloid-β Load Is Related to Worries, but Not to Severity of Cognitive Complaints in Individuals With Subjective Cognitive Decline: The SCIENCe Project
Source: Front Aging Neurosci. 2019 Jan 25;11:7. doi: 10.3389/fnagi.2019.00007 (PMC6362417; doi:10.3389/fnagi.2019.00007)
Supplement: Supplementary file 1 [file Table_1.DOCX]

**Supplementary table S1**. Statistical models for memory deficit self-awareness indexes

| **Memory Deficit**  **Awareness indexes** | **Variables** | **Standardized betas**  **(p-values)** |  |
| --- | --- | --- | --- |
| **RBMT-based** | Age | 0.21 (0.03) |  |
|  | Sex | 0.12 (0.17) |  |
|  | Education | 0.20 (0.13) |  |
|  | CES-D (depressive symptoms) | -0.25 (0.005) |  |
|  | Mean cortical [^18^F]florbetapir BP_ND_ | 0.55 (<0.001) |  |
|  | Interaction  Education*[^18^F]florbetapir BP_ND_ | -0.58 (0.002) |  |
|  |  |  |  |
| **RAVLT-based** | Age | -0.06 (0.53) |  |
|  | Sex | 0.00 (0.99) |  |
|  | Education | 0.14 (0.31) |  |
|  | CES-D (depressive symptoms) | -0.31 (0.001) |  |
|  | Mean cortical [^18^F]florbetapir BP_ND_ | 0.38 (0.01) |  |
|  | Interaction  Education*[^18^F]florbetapir BP_ND_ | -0.42 (0.03) |  |
|  |  |  |  |
